# Essential Oil From *Dittrichia viscosa* L.: A Potential Source of Bioactive Substances With Antioxidant, Antimicrobial, and Antidiabetic Properties: *In Vitro* and *In Silico* Studies

**DOI:** 10.1155/adpp/9951847

**Published:** 2025-08-18

**Authors:** Nesrine Benkhaira, Mohamed El Fadili, Naoufal El Hachlafi, Rhizlan Abdnim, Saad Ibnsouda Koraichi, Kawtar Fikri-Benbrahim

**Affiliations:** ^1^Laboratory of Microbial Biotechnology and Bioactive Molecules, Faculty of Sciences and Technologies, Sidi Mohamed Ben Abdellah University, Fes, Morocco; ^2^LIMAS Laboratory, Faculty of Sciences Dhar El Mahraz, Sidi Mohamed Ben Abdellah University, Fes, Morocco; ^3^Faculty of Medicine and Pharmacy, Ibn Zohr University, Guelmim 81000, Morocco; ^4^Laboratory of Bioresources, Biotechnology, Ethnopharmacology, and Health, Faculty of Sciences, Mohammed First University, Oujda, Morocco

**Keywords:** ADME, antidiabetic, antimicrobial, antioxidant, *Dittrichia viscosa*, docking, phytochemical

## Abstract

In Morocco, *Dittrichia viscosa* L. has long been used to treat a variety of illnesses. The objective of this work was to comprehensively evaluate the essential oil (EO) derived from *D. viscosa* essential oil (DVEO) for its antibacterial, antidiabetic, and antioxidant effects and to confirm the in vitro results using in silico approaches. The chemical composition of DVEO was investigated using gas chromatography–mass spectrometry (GC–MS). The antioxidant ability was evaluated using the 2,2-diphenyl-1-picrylhydrazyl (DPPH) assay and the β-carotene bleaching inhibitory activity. To assess the antibacterial potential, disc diffusion and in vitro microdilution were employed. The antidiabetic potential of DVEO was further investigated in this study by evaluating its inhibitory effects on α-amylase and α-glucosidase enzymes. The molecular docking was employed to support the experimental findings by modeling interactions between key DVEO compounds and relevant protein targets. Pharmacokinetics and toxicity were evaluated and predicted for these compounds. GC–MS analysis revealed that Shyobunol constituted over 40% of the DVEO composition. In the β-carotene and DPPH tests, DVEO exhibited a notable antioxidant effect, with IC_50_ values of 28.93 ± 0.37 μg/mL and 759.44 ± 4.35 μg/mL, respectively, compared with standard antioxidant BHT (19.23 ± 0.53 μg/mL). The EO also demonstrated strong antibacterial activity, especially against *Staphylococcus aureus* (inhibitory zone [IZ] = 17.11 ± 1.11 mm) and *Bacillus subtilis* (IZ = 22.05 ± 0.98 mm). By inhibiting intestinal α-glucosidase and pancreatic α-amylase, the DVEO had IC_50_ values of 0.341 ± 0.11 mg/mL and 0.361 ± 0.04 mg/mL. The IC_50_ was determined to be between 0.281 and 0.401 mg/mL based on replicate experiments. Molecular docking simulations indicated that DVEO constituents interact favorably with the active sites of key enzymes, reinforcing their potential biological relevance. Several compounds also displayed favorable physicochemical, pharmacokinetic, and toxicity profiles, supporting their role as potent therapy candidates. These findings highlight DVEO as a valuable source of bioactive molecules with potential applications in drug discovery and development.

## 1. Introduction

The emergence of global health threats linked to the increasing spread of antibiotic-resistant bacteria poses a serious challenge, significantly impacting pharmaceutical development due to the lack of effective new antibiotics. The cause includes multidrug-resistant bacteria such as *Acinetobacter baumannii*, *Klebsiella pneumoniae*, *Pseudomonas aeruginosa*, methicillin-resistant *Staphylococcus aureus* (MRSA), some Enterobacteriaceae, and other microorganisms on its global priority pathogen list to which the World Health Organization (WHO) responded [1]. The WHO has called for its commitment to support research on methods for combating these infections, including fungal infections, to become a common health concern for otherwise healthy individuals as well as those with weakened immune systems [2].

In addition, diabetes mellitus (DM), a chronic condition characterized by persistently elevated blood glucose levels, has emerged as a major metabolic disorder contributing significantly to global morbidity and mortality. This begins with inadequate insulin production or dysfunctional insulin, which is usually due to one of the many factors including obesity, sedentary lifestyle, or oxidative stress [3]. High glucose levels stimulate the formation of ROS, which in turn evoke many pro-oxidizing pathways like polyol, hexosamine, PKC, and AGE, promoting cellular damage [4].

The aforementioned health concern has made plants an important source of bioactive compounds with useful pharmaceutical properties. In this sense, plant metabolite searching revolving around their biological activities is still an important scientific problem that impacts natural bioactive compound discovery and facilitates the establishment of green technologies [2].

Traditional medicine has been an integral part of human life throughout human history, and medicinal plants have been used to treat numerous illnesses and conditions [5]. An example is *Dittrichia viscosa*, previously named *Inula viscosa*, a perennial shrub from the Mediterranean. Medicinally, this plant has been used in Mediterranean folk medicine for its antimicrobial, antipyretic, and anti-inflammatory properties [6]. Previously, it has been demonstrated that the bioactive compounds of *D. viscosa* including terpenoids, sesquiterpene lactones, and flavonoids [7] have properties of acting as antioxidant [8], anti-inflammatory [9], antimicrobial [10], and antiproliferative [11].

Although widely used, there is a lack of studies focusing on the biological activities exhibited by essential oils (EOs) obtained from *D. viscosa* species. In fact, EOs are complex mixtures mainly composed of a variety of volatile compounds, including mono- and sesquiterpenes, as well as aromatic and aliphatic compounds derived from phenol [12]. EOs are celebrated for their wide range of beneficial properties as natural additives in foods and fragrances as well as for their therapeutic qualities.

Besides the already well-known antibacterial [13], antifungal [14], and antidiabetic effects [15], they possess a variety of further health-promoting properties. In addition, several EOs have demonstrated antioxidant activity [16]. The present work aims to expand the current knowledge by providing a detailed profiling of the phytochemical composition of *D. viscosa* essential oil (DVEO) and further investigating its antidiabetic potential through the inhibition of α-amylase and α-glucosidase enzymes. In addition, the antimicrobial and antioxidant activities of DVEO will be evaluated. Complementary computational studies, including molecular docking and absorption, distribution, metabolism, excretion, and toxicity (ADMET) analysis, are also conducted to explore the interactions and pharmacokinetics of DVEO compounds. Our goal is to provide a comprehensive biological profiling of DVEO to support its ethnopharmacological applications and to offer multiple directions for future targeted research.

## 2. Materials and Methods

### 2.1. Plant Collection and EO Extraction

The aerial parts of *D. viscosa* were collected in September 2024 from Sefrou, Morocco (33° 49′ 54.7″ N, 4° 49′ 40.8″ W). The plant material was taxonomically identified by a botanist at the Botany Department, Sidi Mohamed Ben Abdellah University, Fez, Morocco, and a voucher specimen was deposited in the university herbarium under the reference BLMUP-350.

**Figure 1:**
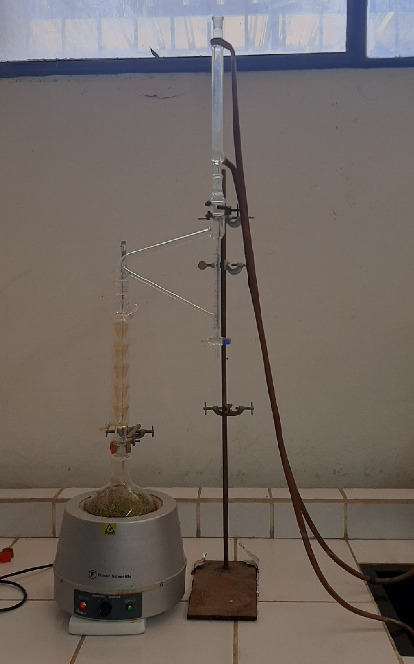
Modified Clevenger-type apparatus used for essential oil extraction.

After collection, the plant material was air-dried in the shade at ambient room temperature (25°C) for 10 days and then manually crushed prior to extraction. EO extraction was carried out using a modified Clevenger-type apparatus (Figure 1), which included a condenser extension to minimize loss and ensure better recovery of volatiles. For each extraction, 650 g of dried plant material was hydrodistilled with 3L of distilled water for 3 h, and the resulting oil was collected by decantation and dried over 5 g of anhydrous sodium sulfate (Na_2_SO_4_) for 2 h to remove any residual moisture. The oil was then stored in amber glass vials at 4°C in the dark until further analysis. Three extractions were done independently, and the average yield, as well as the standard deviation (SD), was calculated.

### 2.2. Chemical Analysis of DVEO

The DVEO's chemical analysis was examined by GC–MS utilizing an Agilent HP 6890 gas chromatography equipped with an HP5973, MS, and capillary column HP-5MS, in accordance with the procedure outlined by [17]. The carrier gas is helium, and the temperature program began at 50°C for 5 minutes before ramping up to 200°C at a rate of 4°C/min. The injector and detector were kept at 250°C, and then, 1 μL of the diluted EO was injected. The MS conditions are as follows: ionization voltage 70 eV, scanning in the range of 35–450 m/z; identification of the components was performed by matching their MS spectra with the data of the NIST/NIH library, after normalizing their peak areas for quantification.

### 2.3. Antioxidant Potential of DVEO

#### 2.3.1. 2,2-Diphenyl-1-picrylhydrazyl (DPPH) Assay

The method described in [18] was used to evaluate the scavenging capacity of DVEO. In short, a 0.001% DPPH solution was mixed with varying quantities of oil, vortexed, and allowed to sit at room temperature for half an hour. BHT was then used as a positive control to assess the absorbance at 517 nm. Every test was conducted three times in parallel, and the scavenging activity was determined by comparing the absorbance of the samples with the control.

#### 2.3.2. β-Carotene Bleaching Test

The procedure outlined in [19] was used to create the emulsion of β-carotene, linoleic acid, and Tween-80. To do this, 500 μL of EOs in various concentrations was combined with 2 mL of this emulsion. OD was measured at 470 nm and compared with a standard antioxidant (α-tocopherol) and a methanol blank. Each test was conducted in triplicate. Antioxidant activity was evaluated as the percentage of residual color according to the following equation:

(1) $$\begin{array}{c} \text{residual color}\left(\%\right)=\left[1-\frac{{\text{OD}}_{\left(0\right)}-{\text{OD}}_{\left(t\right)}}{{\text{OD}}_{\left(0\right)}-{\text{OD}}_{\left(t\right)}}\right]\times 100, \end{array}$$

where OD_(0)_ represents the initial optical density of the EOs and the control and OD_(t)_ corresponds to the optical density of the EOs and the control after 2 h.

### 2.4. Antimicrobial Properties of DVEO

#### 2.4.1. Microbial Strains

Five microbial strains in all were employed: two Gram-negative (Gram^−^) bacteria, *Salmonella enterica* (clinical isolate) and *Escherichia coli* ATCC 25922, one fungal strain, *Candida tropicalis* (clinical isolate), and two Gram-positive (Gram^+^) bacteria, *Staphylococcus aureus* ATCC 29213 and *Bacillus subtilis* ATCC 6633. The microbes came from the Faculty of Sciences' Laboratory of Microbial Biotechnology and Bioactive Molecules in Fez, Morocco.

#### 2.4.2. Disc Diffusion Assay

With certain changes as previously mentioned, the antibacterial activity of DVEO was assessed using the agar disc diffusion method [18]. In short, bacterial culture suspensions were added to yeast extract–peptone–dextrose agar (for Candida) and LB agar (for bacteria). After being soaked in 12 μL of pure EO, sterile paper discs were put on the agar plates. Fluconazole (10 μg/disc) and amoxicillin (10 μg/disc) were employed as standards for fungi and bacteria, respectively. For 24 h, microbial cultures were incubated at temperatures between 30°C and 37°C. The inhibitory zone (IZ) sizes were measured in millimeters following incubation. Based on three separate experimental repetitions, the data were presented as the mean value ± standard error of the mean.

#### 2.4.3. Minimum Inhibitory Concentration (MIC) Assay

With minor adjustments, a previously published technique was used to determine this test. DVEO was produced in Mueller–Hinton broth in serial two-fold dilutions ranging from 8.0% to 0.0625% (v/v) [20]. A total of 100 μL of the serially diluted DVEO, 95 μL of sterile LB broth, and 50 μL of a standardized bacterial suspension were added to each well. Both positive and negative controls were present, with the exception of the bacterial solution. Each well received 15 μL of resazurin after the allotted incubation time, and the wells were then incubated for an additional 30 min. The development of a purple-red color indicated microbial growth. The MIC was defined as the lowest concentration of DVEO that inhibited microbial growth after 24 h for bacteria or 48 h for Candida [19].

#### 2.4.4. Minimum Bactericidal Concentration (MBC) and Minimum Fungicidal Concentration (MFC) Assessment

The test dilutions were subcultured on the LB agar medium in order to measure the MBC and MFC. For bacterial strains, these plates were incubated for 24 h, while for Candida, they were incubated for 48 h. The lowest concentration at which there were no discernible bacterial colonies on the plates was determined to be the MBC or MFC. The MFC/MIC and MBC/MIC ratios were also computed [21].

### 2.5. Antidiabetic Properties of DVEO

#### 2.5.1. In Vitro α–Glucosidase Inhibition

A modified method of [22] was used to evaluate the α-glucosidase inhibitory activity of DVEO. The reaction mixture contained α-glucosidase (0.5 units/mL), phosphate buffer (0.1 M, pH 6.9), and EO at different concentrations (0.1–0.5 mg/mL). The reaction was started with p-nitrophenyl-α-D-glucopyranoside (5 mM) and then incubated for 15 min at 37°C. Acarbose was used as a positive control, and negative and blank controls were also prepared. Sodium carbonate (0.2 M) was added to stop the reaction, and absorbance was measured at 405 nm. To calculate the IC_50_ values, the following equation was used to plot inhibition percentages against EO concentrations:

(2) $$\begin{array}{c} \text{inhibitory activity }\%=\text{Abs}\left(\text{control }500\text{ nm}\right)-\frac{\text{Abs}\left(\text{sample }500\text{ nm}\right)}{\text{Abs }\left(\text{control }500\text{ nm}\right)}\times 100, \end{array}$$

where Abs_Control 500 nm_: absorbance of the control without sample and Abs_Sample 500 nm_: absorbance of sample (EO or acarbose).

#### 2.5.2. In Vitro α-Amylase Inhibition

Using the 3,5-dinitrosalicylic acid (DNSA) assay, the α-amylase inhibition potential was evaluated [19]. A stock solution of DVEO (1 mg/mL) was prepared in phosphate buffer with NaCl (pH 6.9) and serially diluted to concentrations of 0.1–0.5 mg/mL. Each dilution was combined with α-amylase (2 units/mL) and incubated at 30°C for 10 min, after which a starch solution (1%) was added and the reaction was stopped using sodium potassium tartrate in NaOH and DNSA. The solution was then cooled, and its absorbance was measured at 540 nm.

A blank (without enzyme) and acarbose as a positive control were included. IC_50_ values were established by graphing inhibition percentages against EO concentrations.

### 2.6. In Silico Predictions of Physicochemical and Pharmacokinetic Features

Based on the identification of the chemical composition of DVEO by gas chromatography–mass spectrometry (GC–MS) analysis, Shyobunol was determined to be the major constituent, exhibiting the highest peak area (41.64%). Following this characterization, in vitro assays were conducted on *D. viscosa*, and complementary in silico studies were performed to predict its physicochemical properties and pharmacokinetic profiles, including ADMET [23, 24]. In addition, the compound was evaluated using the BOILED-Egg model and bioavailability radar via the PKCSM platform and the SwissADME online server, to further assess its pharmacokinetic behavior and drug-likeness properties [24–26].

#### 2.6.1. In Silico Molecular Docking

In addition to prior computational analyses, molecular docking simulations were performed to evaluate the interaction of the investigated compound with the selected target proteins [25, 27]. The aim was to explore its potential inhibitory mechanisms by analyzing specific intermolecular interactions that may support the EO's reported antioxidant [26], antibacterial [28], and antidiabetic [17] activities. Four target proteins were selected based on their biological relevance and structural availability in the Protein Data Bank (PDB): NAD(P)H oxidase (PDB ID: 2CDU; resolution: 1.80 Å), a water-forming oxidoreductase from *Lactobacillus sanfranciscensis* involved in redox homeostasis; DNA gyrase subunit B (PDB ID: 6F86; resolution: 1.90 Å), an isomerase from *Escherichia coli* and a well-established antibacterial target; human salivary α-amylase (PDB ID: 1SMD; resolution: 1.60 Å), an O-glycosyl hydrolase essential for carbohydrate digestion; and lysosomal acid α-glucosidase (PDB ID: 5NN5; resolution: 2.00 Å), a human hydrolase critical for glycogen degradation and glucose metabolism.

Protein structures were obtained from the RCSB PDB and preprocessed by removing cocrystallized ligands, water molecules, and heteroatoms. Polar hydrogens were added, and Gasteiger partial charges were assigned using AutoDockTools-1.5.6. Energy minimization was applied as needed to resolve steric clashes. Active sites were defined based on the locations of native ligands or conserved catalytic residues. Using a grid spacing of 0.375 Å, the grid boxes were centered at specific Cartesian coordinates for each protein target as follows: (*X* = 10.201, *Y* = 0.657, *Z* = 6.149) for 2CDU.pdb, (*X* = 67.315, *Y* = 31.922, *Z* = 54.435) for 6F86.pdb, (*X* = 8.366, *Y* = 58.678, *Z* = 19.069) for 1SMD.pdb, and (*X* = 1.746, *Y* = −26.552, *Z* = 87.32) for 5NN5.pdb. Finally, docking simulations and subsequent visualization of binding affinities and molecular interactions between Shyobunol and the target proteins were performed using a combination of AutoDockTools-1.5.6 and Discovery Studio 2021 [29, 30].

### 2.7. Statistical Analysis

All results are expressed as mean ± SD from three independent experiments. Statistical analysis was performed using GraphPad Prism 9.0, and differences between means were assessed using one-way ANOVA followed by Tukey's multiple comparison test. A *p* value < 0.05 was considered statistically significant.

## 3. Results and Discussion

### 3.1. Chemical Composition of DVEO

In our study, the EO yield from the aerial parts of *D. viscosa* collected in Sefrou, Morocco, was 2.45 ± 1.15% (v/w), being much higher than those reported for other climes. For instance, Jerada et al. had got a yield of 0.085% from samples collected in southeast Morocco [31], whereas Eddardaki et al. and Vuko et al. did not discuss yields but mostly underlined the low content attributed to this species [6, 32]. The observed variation can be explained by altitudinal, edaphic, and climatic factors in the Sefrou area, as well as by the presence of chemotypes, harvesting time, and extraction parameters of the plant material used. Additional factors that would contribute to the more efficient extraction of the EO might be the freshly dried plant material used in our extraction and the extended hydrodistillation period. Other researchers working on EOs have reported similar yield fluctuations, indicating how regional and methodological parameters influence the yield of the EO [33].

**Figure 2:**
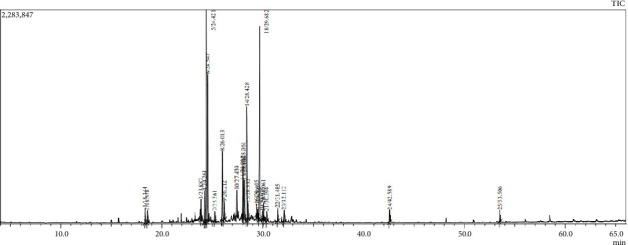
GC-MS total ion chromatogram (TIC) of *Dittirchia viscosa* essential oil (DVEO) showing the retention times of identified compounds.

The GC–MS analysis was conducted for DVEO by combining the retention time and mass spectrometry data in order to identify the components of DVEO (Figure 2). The results are listed in Table 1, describing the identified molecules, along with their percentages, RT, and molecular weight and formula. Thus, 99.43% of the identified compounds were elucidated, and only 0.57% remained unidentified trace elements. Indeed, 20 components from various terpenoid chemical families were identified (Table 1). Shyobunol was detected in the highest amount with a percentage of 41.64%, followed by α-bisabolol (12.78%), τ-muurolol (10.49%), and D-germacren-4-ol (10.09%). These compounds were mainly belonging to the oxygenated sesquiterpenes group (91.44%).

DVEO has also been analyzed for its chemical composition. Mssillou and his colleagues in 2022 unveiled other chemotypes: bornyl acetate (40.00%) and borneol (9.30%) in DVEO collected from the Fez region. In Algerian DVEO, as well, a different chemotype was identified. These were dominated by different compounds: for example, 12-carboxyeudesma-3,11(13) diene (29.00%), linolenic acid (7.80%), and pentacosane (5.40%) [34]. Another diverse chemotype was dominated by isocostic acid (59.00%) and fokienol (14.60%) as reported by the other study [35]. Furthermore, the studies conducted in *D. viscosa* from Spain have reported the major constituents as borneol (25.00% and 20.90%) and bornyl acetate (20.00% and 50.00%) from two diverse EO samples [36]. Such diversities on the chemical profile would indicate that *D. viscosa* from different regions or conditions can produce EOs with significantly different compositions, leading to the classification of distinct chemotypes.

### 3.2. Antioxidant Activity of DVEO

Recent studies have pointed out the high antioxidant activity of several EOs, raising great interest in their possible uses as natural preservatives, health-promoting agents, and active ingredients for cosmetic and pharmaceutical formulations. In fact, EOs may exert their action as natural antioxidants or coadjutants to synthetic antioxidants against multiple mechanisms such as free radical scavenging, lipid oxidation inhibition, and cellular oxidative stress mitigation [37, 38]. Here, we assessed the antioxidant potential of DVEO using two commonly used in vitro tests, namely, the DPPH and β-carotene assays.

Indeed, according to Tables 2 and 3, the DVEO has shown important antiradical effect, represented by its capacity to scavenge free radicals (IC_50_ = 28.93 ± 0.37 μg/mL) when compared with standard antioxidant BHT (19.23 ± 0.53 μg/mL) used as a reference point, while statistically significant at *p* < 0.05. Furthermore, for the first time, the antioxidant activity of DVEO was also undertaken in this study using the β-carotene assay. These results suggest that DVEO possesses promising preventive activity against lipid peroxidation, manifested by the reduced discoloration of the β-carotene solution over time, hence giving an IC_50_ value of 759.44 ± 4.35 μg/mL, though less effective compared with the synthetic antioxidant α-tocopherol with an IC_50_ of 113.09 ± 1.15 μg/mL.

The literature presents that DVEO exerts different IC_50_ values, depending on the extraction technique, plant origin, and part of the plant used. For instance, the IC_50_ value in the DPPH assay was 1.30 ± 0.05 mg/mL for DVEO from leaves, indicating very strong radical-scavenging action [8]. Higher IC_50_ values for DVEO from various plant parts (leaves, flowers, and aerial parts) ranged from 9.25 to 9.75 mg/mL, according to another study [39]. This suggests that the antioxidant capacity can vary greatly depending on the particular sample and the extraction and testing conditions. DVEO also showed interesting antioxidant potential using other in vitro tests. In fact, through the phosphomolybdenum test, DVEO from leaves showed total antioxidant activity in the range of 39.81 ± 0.7–192.1 ± 0.8 mg AAE/g while DVEO from the aerial part had an EC50 of 36.0 ± 2.5 mg/mL in the FRAP assay [8].

Generally, the biological activities of DVEO were compared with the standard reference compounds to assess its therapeutic potential. In the DPPH assay, DVEO exhibited a scavenging activity with an IC_50_ of 28.93 ± 0.37 μg/mL, which, while slightly lower than BHT (IC_50_ = 19.23 ± 0.53 μg/mL), still indicates strong radical-scavenging capacity. In the β-carotene bleaching assay, DVEO was less potent (IC_50_ = 759.44 ± 4.35 μg/mL) compared with α-tocopherol (IC_50_ = 113.09 ± 1.15 μg/mL), suggesting a moderate protective effect against lipid peroxidation.

Its potential use in the preservation of foods and as a biopharmaceutical agent comes from the general antioxidant properties observed in DVEO. Neutralization of the free radicals results because several kinds of bioactive compounds are present to help in keeping away from some oxidation-related diseases. Although various studies support the antioxidant efficacy of DVEO through different methodologies, further research is required to be able to clearly define the structure–activity relationships and optimize extraction conditions for the maximal exploitation of this natural source for its antioxidant potential.

### 3.3. Antimicrobial Activity of DVEO

In this study, diffusion and microdilution assays were carried out to assess the antibacterial properties of DVEO (Tables 4 and 5). The EO displayed potent antibacterial and antifungal activities against all pathogens tested that were comparable to those of standard drugs fluconazole and amoxicillin (ANOVA, *p* < 0.05).

The inhibition zones show that *Bacillus subtilis* (22.05 ± 0.98 mm), *Salmonella enterica* (15.11 ± 0.75 mm), *Staphylococcus aureus* (17.11 ± 1.11 mm), and *Escherichia coli* (14.52 ± 0.08 mm) were the bacteria that showed the least resistance to the EO under investigation. The disc diffusion method showed that DVEO had a significant effect against *Candida tropicalis* (16.05 ± 0.5 mm) that was comparable to fluconazole (ANOVA, *p* < 0.05).

The results of the MIC, MBC, and MFC assays reiterated the disc-diffusion test results (Table 5). The MIC numbers showed that the lowest concentration of the DVEO affecting the visible growth of the bacteria and fungi ranged from 0.125% to 2% (v/v). Likewise, the MBC and MFC found that concentrations of EO from 0.5% to 2.0% (v/v) are the lowest concentrations that will kill bacteria and kill fungus cells, respectively. The ratios of MBC/MIC and MFC/MIC from 1.0% to 4.0% (v/v) suggest that the EO could be bactericidal and fungicidal and will be discussed further.

These findings add weight to previous studies on *D. viscosa* extracts for the antimicrobial activities, notably with respect to a similar study from Morocco that stated that EO from *D. viscosa* flowers coming from Northern Morocco was very potent against *E. coli* (9.5 ± 0.5 mm), *S. aureus* (31.0 ± 1.5 mm), *Candida albicans* (20.4 ± 0.5 mm), and *S. cerevisiae* (28.0 ± 1.0 mm) exhibiting MIC between 0.125% and 2% (v/v) [8].

Blanc et al. [40] described *D. viscosa* collected from France, for two separate biologically active EO components, neutral and acidic fractions, against bacteria, fungi, and yeasts. From their data, the neutral part tested was inactive against all microorganisms, while the acidic part worked against the rest except *Escherichia coli*. *Escherichia coli* was active at 2.5 μL/mL against *Staphylococcus aureus*, *Cryptococcus neoformans*, and *Cladosporium cladosporioides*. DVEO maintained a potent effect against *Staphylococcus epidermidis*, *Streptococcus faecalis*, and *Proteus vulgaris* (MIC = 1.25 μL/mL).

However, Silva et al. [41] showed that the aerial parts' EO of the Portuguese *D. viscosa* is not effective against *Listeria monocytogenes*, while it completely inhibited the *Helicobacter pylori* growth at 88.80–133.20 μg·mL^−1^.

A recent Moroccan study demonstrated that EOs extracted from the leaves, stems, and flowers of *D. viscosa* exhibit significant antifungal activity against *Candida albicans*, *Aspergillus niger*, and *Trichophyton rubrum*. Notably, the EOs showed the most favorable minimum fungicidal concentrations for these human pathogenic fungi, ranging from 1.88 to 3.35 μL/mL, compared with *Fusarium oxysporum*, which had an MFC of 3.5 μL/mL [32].

In addition, the antibacterial activity exhibited by DVEO in the present study, particularly against *S. aureus* (IZ = 17.11 ± 1.11 mm) and *B. subtilis* (IZ = 22.05 ± 0.98 mm), aligns with findings from other medicinal plants known for their antimicrobial potential. For example, the EO of *Thymus serpyllum* showed significant antibacterial activity with inhibition zones ranging from 13.66 ± 0.58 mm to 33.66 ± 1.52 mm against various bacterial strains, largely attributed to its high thymol content [42] Similarly, the chloroform subfraction of *Rheum emodi* demonstrated strong antibacterial activity against *S. aureus*, *K. pneumoniae*, and *E. coli*, with MIC values as low as 1.95 μg/mL [43]. These findings support the effectiveness of plant-derived EOs as potent antibacterial agents. While the chemical profiles differ, with DVEO being rich in Shyobunol and *T. serpyllum* in thymol, the comparable biological activities suggest that structurally diverse phytocompounds can exert significant antimicrobial effects [44]. Furthermore, these results collectively highlight the growing interest in exploring EOs not only as standalone antibacterial agents but also as synergistic enhancers of conventional antibiotics [45].

DVEO contains a significant proportion of oxygenated sesquiterpenes, which is probably why our study found such strong antibacterial action. It is significant for the quantity of Shyobunol in the oil. Also, possibly, the wide-spectrum efficacy of DVEO is due to the synergistic interactions among the different components of its formulation [46]. However, it should be noted that the activity of the oil against a wide range of microbial strains was not tested in this study. For this reason, we recommend extensive research so that the further understanding of its effects against a wider variety of microorganisms can be undertaken.

### 3.4. Antidiabetic Activity of DVEO

**Figure 3:**
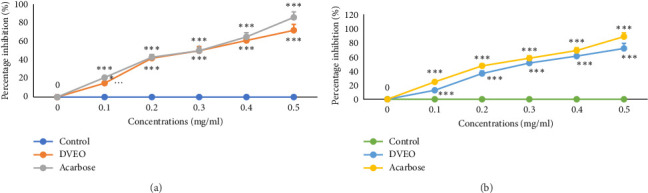
Inhibitory effect of α-amylase (a) and α-glucosidase (b) enzymes by DVEO and acarbose in vitro. The values are the means ± SEM (*n* = 3). ^∗∗^*p* < 0.01 and ^∗∗∗^*p* < 0.001 as functions of acarbose.

Results showed that DVEO significantly reduced intestinal α-glucosidase and pancreatic α-amylase enzymatic activities in a concentration-dependent manner (*p* < 0.001) (Figure 3), with IC_50_ values of 0.361 ± 0.04 and 0.341 ± 0.11 mg/mL, respectively (Table 6). DVEO's α-amylase and α-glucosidase enzyme inhibitory actions were marginally similar to those of acarbose (IC_50_ = 0.281–0.401 mg/mL).

DM is characterized by abnormal glucose metabolism that imparts disturbances to insulin synthesis in pancreatic cells [3]. Carbohydrate absorption through the gastrointestinal tract is an essential mechanism for normal blood glucose homeostasis. Enzymes such as α-amylase and α-glucosidase have a direct role in the conversion of complex sugars into simpler ones. With the inhibition of these two enzymes being an equally important natural product management approach for hyperglycemia, a great deal of effort has gone into their study in recent times [47]. Therefore, this work aimed to evaluate the inhibitory activities of *D. viscosa* against α-amylases and α-glucosidases.

To our knowledge, there are no studies on the possible antidiabetic effects of DVEOs; however, some studies have investigated the antidiabetic properties of its extracts. These investigations have demonstrated that by blocking the α-amylase and α-glucosidase enzymes, *D. viscosa* extracts have encouraging antidiabetic potential [48].

A recent study used a rat model of high-fat diet (HFD)/streptozotocin (STZ)-induced diabetes to investigate the in vivo antidiabetic benefits of gold nanoparticles (AuNPs) made from Jordanian *Dittrichia viscosa* leaf extract. When compared with the untreated diabetic group, treatment with AuNPs dramatically decreased blood glucose levels, gene expression, and the activity of hepatic phosphoenolpyruvate carboxykinase (PEPCK), a crucial lyase enzyme in gluconeogenesis (*p* < 0.05). These results imply that AuNPs made from *D. viscosa* leaf extract may reduce hepatic gluconeogenesis by inhibiting PEPCK expression, hence lowering hyperglycemia in HFD/STZ-induced diabetic rats [49].

Another study investigated the potential of extracts from the aerial parts of Moroccan plants to prevent hyperglycemia. When compared with the common medication acarbose, all extracts showed greater α-glucosidase inhibition (IC_50_ = 33.0 μg/mL). The enzyme inhibition percentages varied from 333 μg/mL to 10 μg/mL, with the methanolic extract of *D. viscosa* exhibiting the highest inhibitory activity against α-glucosidase, with an IC_50_ value of 22.3 μg/mL [50]. DVEO demonstrated IC_50_ values of 0.341 ± 0.11 mg/mL and 0.361 ± 0.04 mg/mL for α-amylase and α-glucosidase inhibition, respectively, which are within the range of acarbose (IC_50_ = 0.281–0.401 mg/mL). These results highlight that DVEO exhibits comparable in vitro enzyme inhibition to acarbose, suggesting its potential as a natural therapeutic alternative in diabetes management.

### 3.5. In Silico Results of Physicochemical and Pharmacokinetic Properties

In silico results of physicochemical properties presented in Table 7 confirm that the major compound, namely, Shyobunol was predicted with a good profile justified with molecular weight inferior to 500 g/mol, molar refractivity (MR) index included between 40 and 130, Log P less than 5, and acceptors and donors of hydrogen bonds not exceeding 10 and 5, respectively. Therefore, it was predicted with a good physicochemical profile that meets all five rules of Lipinski.

In addition, the compound under study was equally predicted with a good ADMET pharmacokinetic profile, explained by an excellent human intestinal absorption (HIA of 95.051%), important permeabilities to the blood–brain barrier (BBB), and central nervous system (CNS), potent inhibition to 2C19 cytochrome, with an absence of hepatotoxicity effect on human body. However, it could also produce a positive skin allergy effect on the human body, as resulted in Table 8.

**Figure 4:**
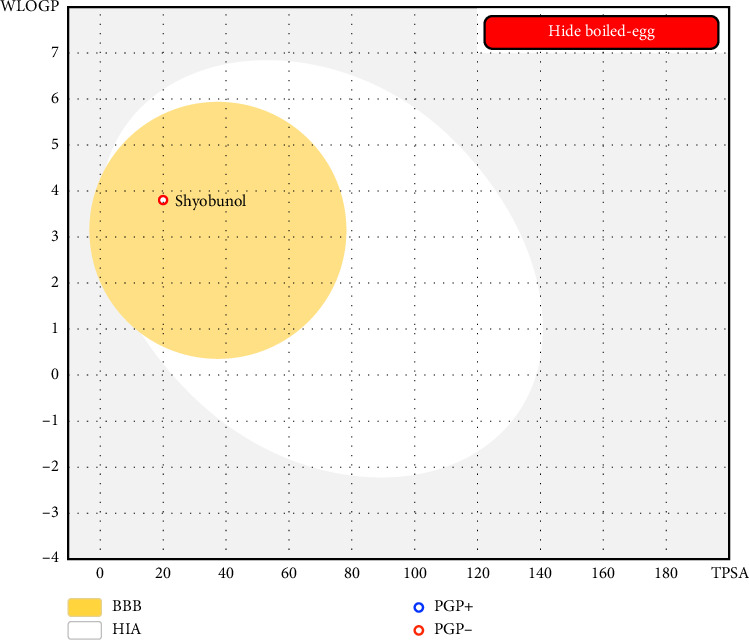
The predictive model of Egan's BOILED-Egg for Shyobunol.

**Figure 5:**
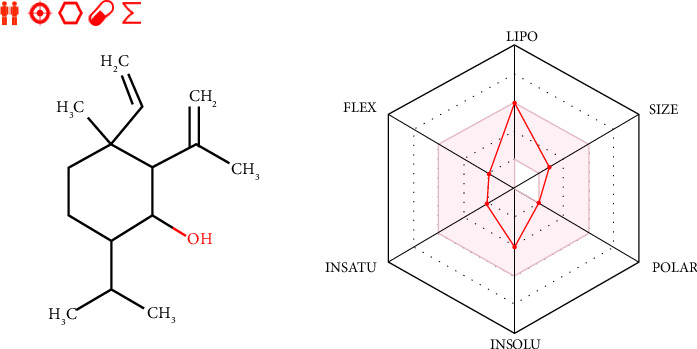
Bioavailability radar of Shyobunol as a major compound of *Dittricha viscosa* essential oil.

The examined compound was part of yellow Egan's BOILED-Egg, so it was predicted to cross the BBB with the highest probability. Then, it was predicted as a potent agent of CNS as shown in the predictive model of Egan's BOILED-Egg (Figure 4). Moreover, the candidate ligand was also predicted to have excellent oral bioavailability since it was predicted with a bioavailability radar around the ideal bioavailability zone highlighted in pink as displayed in Figure 5 [51, 52].

### 3.6. Molecular Docking Simulations

**Figure 6:**
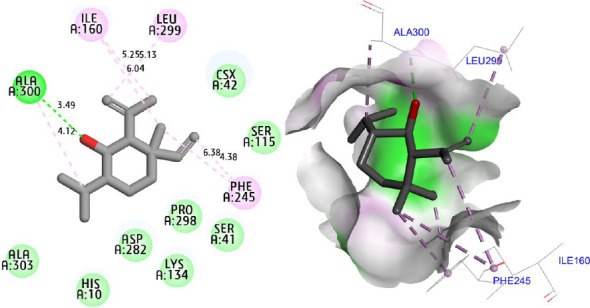
Two- and three-dimensional views of the inhibition mechanism for Shyobunol in the complex with NADPH oxidase protein (2CDU.pdb).

**Figure 7:**
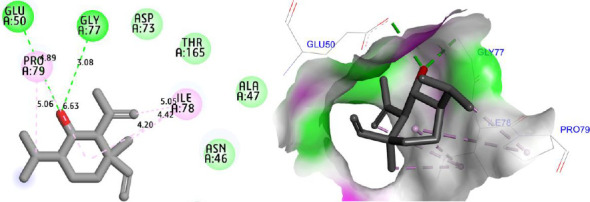
Two- and three-dimensional views of the inhibition mechanism for Shyobunol in the complex with DNA gyrase-B protein (6F86.pdb).

**Figure 8:**
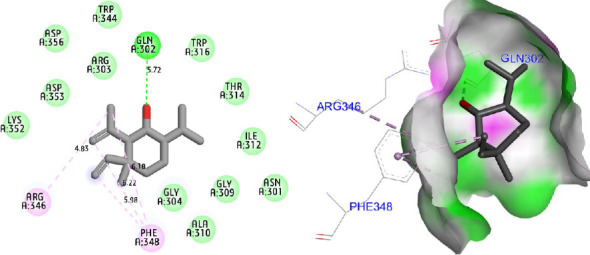
Two- and three-dimensional views of the inhibition mechanism for Shyobunol in the complex with alpha-amylase protein (1SMD.pdb).

**Figure 9:**
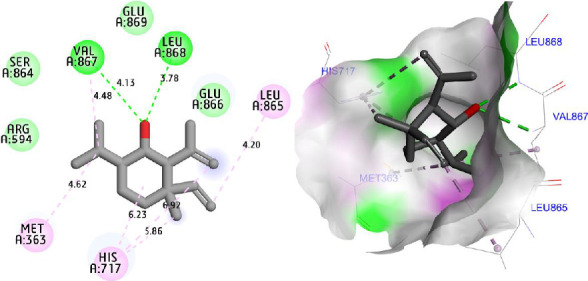
Two- and three-dimensional views of the inhibition mechanism for Shyobunol in the complex with alpha-glucosidase protein (5NN5.pdb).

Molecular docking simulations were carried out to explore the binding affinity and interaction profile of Shyobunol with four selected protein targets involved in antioxidant, antibacterial, and antidiabetic activities, including both α-amylase and α-glucosidase enzymes. The molecular docking results presented in Figure 6 indicate that Shyobunol was first docked to NADPH oxidase protein (2CDU.pdb) with the lowest binding energy of −6.48 kcal/mol, forming one hydrogen bond fixed toward Ala300 amino acid residue (A.A.R), more than alkyl and pi-alkyl bonds detected with Phe245, Leu299, and Ile160 A.A.Rs. Second, the studied compound was equally docked to DNA gyrase-B protein as an antibacterial protein with a binding energy of −5.91 Kcal/mol, forming two hydrogen bonds detected toward Glu50 and Gly77 A.A.Rs, in addition to alkyl and pi-alkyl bonds fixed to Pro79 and Ile78 A.A.Rs (Figure 7). In addition, the major compound was third docked to both antidiabetic proteins of alpha-amylase (1SMD.pdb) and alpha-glucosidase (5NN5.pdb) with largely negative energies of −6.21 and −6.52 kcal/mol respectively, producing one hydrogen bond detected toward Gln302 A.A.R, more than alkyl and pi-alkyl bonds created with Arg346 and Phe348 A.A.Rs against alpha-amylase protein as displayed in Figure 8, more than two hydrogen bonds detected toward Val867 and Leu868 A.A.Rs, in addition to alkyl and pi-alkyl bonds detected toward His717, Met363, and Leu865 A.A.Rs in A chain against alpha-glucosidase protein as presented in Figure 9.

**Figure 10:**
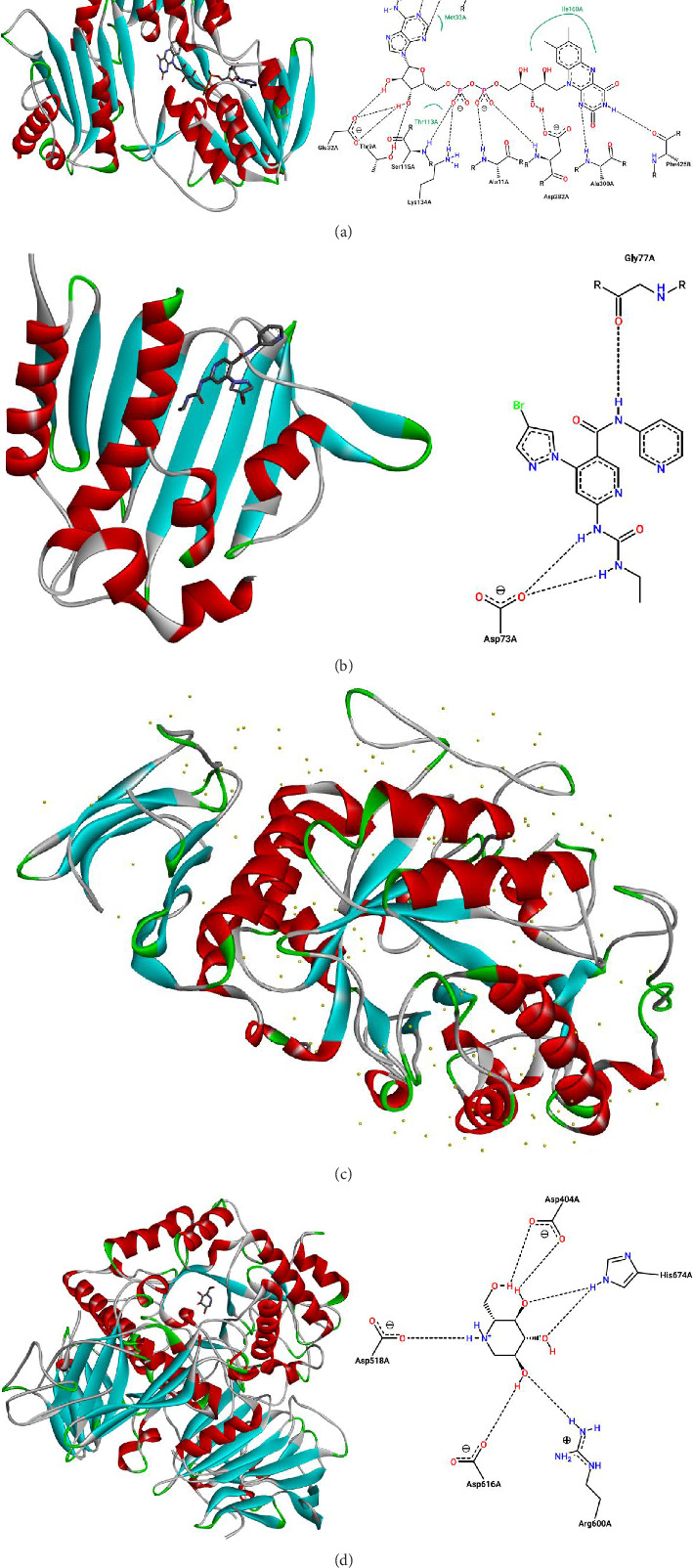
(a) Active sites of NAD(P)H oxidase protein (PDB ID of 2CDU) in the complex with its native ligand (flavin adenine dinucleotide). (b) Active sites of DNA gyrase-B protein (PDB ID of 6F86) in the complex with its native ligand (4-(4-bromo-1H-pyrazol-1-yl)-6-[(ethylcarbamoyl)amino]-N-(pyridin-3-yl)pyridine-3 carboxamide). (c) Unknown active sites of α-amylase protein (PDB ID of 1SMD) in the absence of native and cocrystallized ligand. (d) Active sites of α-glucosidase protein (PDB ID of 5NN5) in the complex with its native ligand (1-deoxynojirimycin).

For comparison, the candidate compound Shyobunol exhibited key intermolecular interactions with low binding energies upon complexation with the selected protein targets. It formed similar interactions to those observed with the native ligand flavin adenine dinucleotide (FAD) of the NADPH oxidase protein (2CDU), notably involving Ala300 and Phe245 active sites, as shown in Figures 10(a), 10(b), 10(c), and 10(d). Likewise, Shyobunol shared common binding features with the cocrystallized ligand [4-(4-bromo-1H-pyrazol-1-yl)-6-[(ethylcarbamoyl)amino]-N-(pyridin-3-yl)pyridine-3-carboxamide] in the DNA gyrase-B structure (5NN5), particularly involving the Gly77 active site. In the case of α-amylase (1SMD), where no cocrystallized ligand was available for direct comparison, Shyobunol established strong and specific interactions with key residues. For α-glucosidase (5NN5), the compound also exhibited typical but slightly different interactions compared with known active site residues, as summarized in Table 9.

Therefore, in silico molecular docking results support the in vitro bioactivities of DVEO. Shyobunol showed good binding affinities to NADPH oxidase (−6.48 kcal/mol), α-amylase (−6.21 kcal/mol), and α-glucosidase (−6.52 kcal/mol), forming key hydrogen bonds at the active sites. These interactions align with the experimental IC_50_ values observed for DVEO in antioxidant (28.93 ± 0.37 μg/mL) and antidiabetic assays (0.361 ± 0.04 and 0.341 ± 0.11 mg/mL), supporting the role of Shyobunol in the observed biological effects.

In this study, Shyobunol exhibited strong binding affinities to antioxidant and antidiabetic targets such as NADPH oxidase, α-amylase, and α-glucosidase, forming multiple hydrogen bonds within the active sites. These interactions imply a direct inhibitory potential that may underlie the observed in vitro effects. Moreover, previous reports on EOs rich in Shyobunol, such as those from *Syzygium cumini, Boswellia dalzielii*, *Cinnamomum travancoricum*, *Pulicaria somalensis*, and *Schinus molle* [47–49], have consistently demonstrated notable antioxidant and antidiabetic activities, supporting Shyobunol's possible role as a bioactive contributor.

## 4. Conclusion

The EO of *D. viscosa* L. contains a variety of bioactive compounds with potential antibacterial, antidiabetic, and antioxidant activities. The oil exhibited a significant antidiabetic potential, with great inhibition of α-amylase and α-glucosidase enzymes, while remarkable antioxidant activity was demonstrated by the DPPH and β-carotene assays. When it comes to antifungal against *Candida tropicalis*, the EO also displayed a strong level of activity, whereas noteworthy activity against *Bacillus cereus*. Such activity was also confirmed by MIC and MBC values, proving that it can eliminate bacteria at different concentrations. The in silico toxicity and pharmacokinetic analysis highlighted a favorable safety profile for Shyobunol. The compound showed no predicted hepatotoxicity, mutagenicity, or carcinogenicity, while demonstrating high oral bioavailability and human intestinal absorption (95.05%). Although a potential for skin sensitization was noted, the overall ADMET profile supports the safety of Shyobunol as a bioactive candidate. Because these encouraging effects were observed, several important topics should be the focus of future research. The molecular mechanisms behind the biological effects of DVEO must be clarified by mechanistic research. Its safety and effectiveness in animal models must also be assessed through in vivo research.

## Figures and Tables

**Table 1:** Chemical composition of DVEO.

No.	Chemical compounds	Formula	Molecular weight (g/mol)	Retention time (min)	% Peak relative area	CAS no.
1	Thymol	C_10_H_14_O	150.22	18.34	0.99	89-83-8
2	Carvacrol	C_10_H_14_O	150.2	18.578	1.86	499-75-2
3	Cyclocopacamphenol	C_11_H_18_O	261.09	29.505	0.64	—
4	ß-Acoradienol	C_15_H_26_O	220.35	31.485	1.44	520-30-3
5	D-Cadinene	C_15_H_24_	204.15	26.112	1.32	483-76-1
6	α-Muurolene	C_15_H_24_	204.35	23.887	1.29	34,896-69-6
7	τ-Cadinol	C_15_H_26_O	222.37	28.005	3.67	14,324-55-9
8	Caryophyllene oxide	C_15_H_24_O	220.35	26.212	1.50	1139-30-6
9	Shyobunol	C_15_H_26_O	220.31	24.421	41.64	18,956-24-6
10	τ-Muurolol	C_15_H_26_O	222.36	28.061	10.49	24,631-99-6
11	Selin-6-en-4-alpha-ol	C_15_H_26_O	222.21	27.439	1.44	14,774-24-6
12	Neointermedeol	C_15_H_26_O	222.37	28.532	1.74	16,166-08-2
13	α-Bisabolol	C_15_H_26_O	284,7	29.405	12.78	23,089-26-1
14	D-Germacren-4-ol	C_15_H_26_O	222.37	26.013	10.09	68,102-96-7
15	α-Muurolene-14-ol	C_15_H_26_O	204.35	24.261	1.44	67,591-24-0
16	6-Isopropenyl-4,8a-dimethyl-decalin-1-ol	C_15_H_26_O	220.35	30.394	0.88	—
17	α-Costol	C_15_H_26_O	222.36	32.112	1.25	68,004-30-0
18	Humulane-1,6-dien-3-ol	C_15_H_26_O	222.37	28.166	3.08	—
19	Phytol	C_20_H_40_O	296.53	42.58	1.09	150-86-7
20	Geranylgeraniol	C_20_H_34_O	290.49	25.261	0.80	1496-30-1

Yield (%v/w)					2.45 ± 1.15	
Total identified					99.43	
Oxygenated sesquiterpenes					91.44	
Monoterpene hydrocarbons					2.47	
Oxygenated monoterpenes					3.63	
Others					1.89	

**Table 2:** IC_50_ values of DVEO by DPPH assay.

EO/standard	IC_50_ ±SD (μg/mL)
DVEO	28.93 ± 0.37^a^
BHT	19.23 ± 0.53^b^

*Note:* Data with the same letter in the same assay indicate a nonsignificant difference by Tukey's multiple range test (ANOVA, *p* < 0.05).

**Table 3:** IC_50_ values of DVEO by β-carotene blenching assay.

EO/standard	IC_50_ ± SD (μg/mL)
DVEO	759.44 ± 4.35^a^
α-Tocopherol	113.09 ± 1.15^b^

*Note:* Data with the same letter in the same assay indicate a nonsignificant difference by Tukey's multiple range test (ANOVA, *p* < 0.05).

**Table 4:** Evaluation of DVEO antimicrobial activity using the disc diffusion test.

Microorganisms	Mean zone of inhibition (mm ± SD)		
	DVEO (12 μL/disc)	Amoxicillin (10 μg/disc)	Fluconazole (10 μg/disc)
*Staphylococcus aureus ATCC 29213*	17.11 ± 1.11^b^	19.23 ± 0.15^a^	NT
*Bacillus subtilis ATCC 6633*	22.05 ± 0.98^b^	24.78 ± 0.21^a^	NT
*Escherichia coli ATCC 25922*	14.52 ± 0.08^b^	16.15 ± 0.01^a^	NT
*Salmonella enterica* (clinical isolate)	15.11 ± 0.75^b^	17.22 ± 0.11^a^	NT
*Candida tropicalis* (clinical isolate)	16.05 ± 0.5^b^	NT	18.02 ± 0.05^a^

*Note:* Data sharing the same letter within the same test indicate no significant difference, as determined by Tukey's multiple range test (*p* < 0.05).

Abbreviations: NT, not tested; SD, standard deviation.

**Table 5:** MIC, MBC, MFC, MBC/MIC, and MFC/MIC values of DVEO.

Bacteria	DVEO			Amoxicillin		
	MIC	MBC	MBC/MIC	MIC	MBC	MBC/MIC
*Staphylococcus aureus* ATCC 29213	0.25	1	4	0.25	1	4
*Bacillus subtilis* ATCC 6633	0.125	0.5	4	0.5	0.5	1
*Escherichia coli* ATCC 25922	2	2	1	2	2	1
*Salmonella enterica* (clinical isolate)	1	2	2	2	2	1

Fungi	**DVEO**			**Fluconazole**		
	**MIC**	**MFC**	**MFC/MIC**	**MIC**	**MFC**	**MFC/MIC**

*Candida tropicalis* (clinical isolate)	1	2	2	1	1	1

*Note:* Fluconazole: used as a standard drug.

Abbreviations: DVEO, *Dittrichia viscosa* essential oil; MFC, minimum fungicidal concentration in % (v/v); MIC, minimum inhibitory concentration in % (v/v).

**Table 6:** IC_50_ values of DVEO and acarbose on pancreatic α-amylase and intestinal α-glucosidase enzymes.

	IC_50_ (mg/mL)	
	α-Amylase	α-Glucosidase
DVEO	0.341 ± 0.11^a^	0.311 ± 0.04^a^
Acarbose	0.361 ± 0.03^b^	0.304 ± 0.02^a^

*Note:* Values are expressed as the mean ± SEM (*n* = 3). Data sharing the same letter within the same test indicate no significant difference (*p* < 0.05).

**Table 7:** Prediction of the physicochemical properties of Shyobunol

**Compounds name**	**Physicochemical properties**					**Lipinski's five rules**
	**MW**	**MR index**	**Log P**	**HBA**	**HBD**	**(No/yes)**
**Rule**	**≤ 500 (g/mol)**	**130 ≥ MR index ≥ 40**	**< 5**	**≤ 10**	**< 5**	

C12	222.37	72.06	3.17	1	1	Yes

**Table 8:** Prediction of the ADME-toxicity pharmacokinetic properties for Shyobunol

**Compounds number**	**A**	**D**		**M**							**E**	**T**	
	**Human intestinal absorption**	**Blood**–**brain barrier permeability**	**Central nervous system permeability**	**Substrate**			**Inhibitor**				**Total clearance**	**Hepatotoxicity**	**Skin sensitization**
				**Cytochromes**									
				**2D-6**	**3A-4**	**1A-2**	**2C-19**	**2C-9**	**2D-6**	**3A-4**			
	**(% absorbed)**	**(Log BB)**	**(Log PS)**	**(No/Yes)**							**Numeric (log mL/min/kg)**	**(No/Yes)**	

C12	95.051	0.618	−2.112	No	No	No	Yes	No	No	No	1.385	No	Yes

*Note:* A: absorption; D: distribution; M: metabolism; E: excretion; T: toxicity.

**Table 9:** Molecular docking results of Shyobunol with selected target proteins, including protein classification, active site residues, key intermolecular interactions, and binding energies (kcal/mol).

Shyobunol complexed to	2CDU.pdb	6F86.pdb	1SMD.pdb	5NN5.pdb
Classification	NAD(P)H oxidase protein (oxidoreductase)	DNA gyrase-B protein (isomerase)	α-Amylase protein (hydrolase)	α-Glucosidase protein (hydrolase)

Active sites	**Ala300**- **Phe245**- Lys134- Thr9- Ala11- Ser115- Glu32- Met33- Val81- Asp282	**Gly77**- Asp73	Unknown active sites	Asp404- Asp518- Asp616- Arg600- His674

Produced intermolecular interactions	**Ala300**- **Phe245**- Leu299- Ile160	**Gly77**- Glu50- Pro79- Ile78	Gln302- Arg346- Phe348	Val867- Leu868- Met363- His717- Leu865

Binding energies (kcal/mol)	**−6.48**	**−5.91**	**−6.21**	**−6.52**

*Note:* Binding energies are expressed in kcal/mol. Bold values represent the most favorable (lowest) binding energies for each target, indicating stronger ligand–protein binding affinity.

## Data Availability

The data will be made available on request.
